# Association between thyroid hormones and diabetic kidney disease in euthyroid type 2 diabetes mellitus patients

**DOI:** 10.3389/fendo.2026.1674977

**Published:** 2026-02-02

**Authors:** Jun Liu, Wenwen Jiang, Jingjing Liang, Xiaozhen Ye, Xinyi Yang, Qi Wang, Min Chen, Hanlu Meng, Qiuyue Shen, Yong Zhong, Jiaqing Shao

**Affiliations:** 1 Department of Endocrinology, Jinling Clinical Medical College, Nanjing Medical University, Nanjing, Jiangsu, China; 2 Department of Endocrinology, Jinling Hospital, Affiliated Hospital of Medical School, Nanjing University, Nanjing, Jiangsu, China; 3 Department of Endocrinology, Nanjing Pu Kou People’s Hospital, Nanjing, Jiangsu, China; 4 Department of Health Medicine, Jinling Hospital, Affiliated Hospital of Medical School, Nanjing University, Nanjing, Jiangsu, China

**Keywords:** blood glucose fluctuations, continuous glucose monitoring, diabetic kidney disease, thyroid function, type 2 diabetes mellitus

## Abstract

**Background:**

Thyroid hormones play an important role in the growth and development of the kidneys. However, there are few reports focusing on the correlation between thyroid hormones and diabetic kidney disease (DKD). The purpose of this study was to explore the association between thyroid hormone levels and DKD in euthyroid patients with type 2 diabetes mellitus(T2DM).

**Methods:**

We conducted a retrospective cross-sectional study among hospitalized T2DM patients, and a total of 559 patients were enrolled in this study. The patients were divided into two groups: 136 in the DKD group and 423 in the non-DKD group. All patients received continuous glucose monitoring (CGM), and glucose variability indices were recorded, including the standard deviation of glucose levels (SD), coefficient of variation of glucose (CV), mean amplitude of glycemic excursions (MAGE), and mean of daily differences in glucose (MODD). Univariate ANOVA, Pearson/Spearman correlation analysis, and multivariate logistic regression analysis were employed to investigate the relationship between thyroid hormones, glucose variability, and DKD.

**Results:**

The DKD group exhibited lower free triiodothyronine (FT3) levels compared to the non-DKD group. In patients with type 2 diabetes, glycemic variability indicators (SD, CV, MODD) showed a significant decreasing trend across FT3 tertiles. Furthermore, FT3 was negatively correlated with SD, CV, and MODD. Multivariate logistic regression analysis revealed that FT3 and thyroid-stimulating hormone (TSH) were related to the presence of DKD.

**Conclusions:**

In euthyroid patients with T2DM, thyroid hormone levels influence glycemic variability. Lower levels of FT3 within the normal range are associated with greater glycemic fluctuations. Additionally, in these patients, FT3 and TSH levels are correlated with the occurrence of DKD. Reduced FT3 levels and elevated TSH levels worked as risk factors for the prevalence of DKD.

## 1 Background

Diabetic kidney disease (DKD), a serious and major chronic complication of diabetes, impacts 30% - 40% of diabetic patients in China and 20% - 30% in the US, and it has been the main cause of end-stage renal disease (ESRD) (1–3). Hyperglycemia, hypertension, and proteinuria are key risk factors for DKD (4, 5). Yet, intensive glycemic control, protein-restricted diets, renin-angiotensin-aldosterone system inhibition, and blood pressure management fail to fully prevent DKD progression in type 2 diabetes mellitus(T2DM). Thyroid function is closely linked to diabetes (6). Many studies have shown that even minor fluctuations of thyroid hormone levels within the normal range can impact the development of DKD (7–9). Subclinical hypothyroidism (SCH) is linked to a higher prevalence of DKD in T2DM (10). Levothyroxine replacement therapy may slow the progression of DKD in T2DM patients with SCH (11). Low T3 levels are associated with inflammation in chronic kidney disease (CKD), suggesting a potential role in renal function decline (12). However, the association between thyroid function in normal range and DKD, and how it may affect DKD, is far from being understood. Therefore, our aim is to investigate the relationship between thyroid hormone levels and DKD in euthyroid T2DM patients, an area where existing studies is limited and inconsistent (13, 14). Additionally, we also explored the connection between thyroid function and glycemic fluctuations in euthyroid T2DM patients.

## 2 Methods

### 2.1 Participants

We conducted a retrospective cross-sectional study among hospitalized T2DM patients via the electronic medical record (EMR) system of Jinling Hospital. Jinling Hospital is a large-scale, modern, comprehensive tertiary hospital in Jiangsu Province, China. It houses a specialized endocrine outpatient clinic and inpatient ward, and our patients come from all regions of the country. The final sample size of 559 patients was derived with the following screening process (1): Initial pool: 1,770 hospitalized T2DM patients (January 2017–December 2022) with complete basic demographic and clinical data (2); First exclusion: 910 patients were excluded because they did not undergo continuous glucose monitoring, a core variable required for analyzing glycemic variability, leaving 860 patients (3); Second exclusion: 301 patients excluded for abnormal thyroid function (FT3 < 3.8 or > 6.0 pmol/L, FT4 < 7.86 or > 14.41 pmol/L, TSH < 0.38 or > 5.33 mIU/L) or missing thyroid function test results, resulting in the final cohort of 559 euthyroid T2DM patients. The inclusion criteria were (1): aged ≥ 18 years (2); previously diagnosed with T2DM according to the 1999 WHO diagnostic criteria (15) (3). euthyroid status, defined as free triiodothyronine (FT3) 3.8-6.0 pmol/L, free thyroxine (FT4) 7.86-14.41 pmol/L, and thyroid-stimulating hormone (TSH) 0.38-5.33 mIU/L (reference ranges validated by the clinical laboratory of the Affiliated Jinling Hospital of Nanjing University). T2DM was diagnosed according to the 1999 WHO diagnostic criteria, which require meeting any one of the following conditions: Fasting plasma glucose (FPG) ≥ 7.0 mmol/L (126 mg/dL) (fasting was defined as no caloric intake for at least 8 hours); 2-hour plasma glucose ≥ 11.1 mmol/L (200 mg/dL) during an oral glucose tolerance test (OGTT) with 75 g anhydrous glucose dissolved in water; Random plasma glucose ≥ 11.1 mmol/L (200 mg/dL) in a patient with classic symptoms of hyperglycemia (e.g., polyuria, polydipsia, unexplained weight loss). According to the KDIGO guidelines, DKD was defined as a urinary albumin-to-creatinine ratio (UACR) ≥ 30 mg/g or an estimated glomerular filtration rate (eGFR) < 60 ml/min/1.73 m², persisting for more than 3 months, after excluding other causes of renal impairment. Exclusion criteria included: other diabetes types, acute infections, acute kidney injury, acute diabetic complications, severe heart disease, urinary tract infection, hematuria, other acute stressors, non-diabetic CKD, ESRD needing dialysis, chronic diseases affecting metabolism or thyroid medication use (e.g., levothyroxine, anti-thyroid drugs), malignancies, pregnancy, or breastfeeding. For participants with inconsistent results between the two initial tests, a third confirmatory test was performed, and the diagnosis was based on the majority of consistent results (≥ 2 positive tests). Relevant examinations (e.g., renal ultrasound, autoimmune antibody testing, urine sediment analysis) were performed as needed to rule out these confounders. Our cohort’s baseline characteristics reflect the tertiary hospital setting: the median HbA1c was 8.7%, which is higher than the community-based T2DM cohort median of 7.8% reported in the China National Diabetes and Metabolic Disorders Study (16). Additionally, 57.4% of our patients received insulin therapy (vs. 30% in community cohorts, and 24.3% had DKD (vs.18% in community settings). The studies were approved by the ethics committee of Jinling Hospital affiliated with Nanjing University (number of approval 2019NZKY-008-08; Date: 11 April 2019). All enrolled participants provided written informed consent prior to undergoing CGM during hospitalization.

### 2.2 Clinical and biochemical measurements

Laboratory indicators were measured via fasting venous blood sampling the morning after an 8 to 10 hour overnight fast upon admission. Clinical data collected included age, height, weight, blood pressure, HbA1c, total cholesterol (TC), triglycerides (TG), high-density lipoprotein cholesterol (HDL-C), low-density lipoprotein cholesterol (LDL-C), thyroid-stimulating hormone (TSH), free triiodothyronine (FT3), free thyroxine (FT4), creatinine, uric acid, 24 hour urine protein, and 24 hour urine albumin. Estimate glomerular filtration rate (eGFR) was calculated by the CKD-EPI creatinine equation (17).

### 2.3 CGM parameters

All patients were monitored using the CGM system manufactured by Meiqi Company (Shenzhen, China). To ensure reliable glycemic variability calculations, strict CGM data quality control criteria were applied. Total monitoring duration: Only CGM recordings with a valid monitoring time of ≥ 3 days were included. This threshold was chosen based on recommendations from the International Consensus on Continuous Glucose Monitoring (18), which specifies that ≥48 hours of continuous data are required to capture both intra-day and inter-day glycemic fluctuations, we extended this to 3 days to further reduce random measurement error from short-term deviations (e.g., transient meal-related spikes). Calibration protocol: During the CGM wear period, participants performed an average of 6 fingerstick blood glucose measurements per day (using a standard glucose meter, model: Roche Accu-Chek Performa) at predefined time points (fasting, 2 hours post-breakfast, 2 hours post-lunch, 2 hours post-dinner, bedtime, and one random time). A minimum of 2 valid fingerstick values (glucose range: 3.0–20.0 mmol/L, to avoid calibration bias from extreme values) were entered into the CGM device daily for calibration, in accordance with the manufacturer’s standard operating procedure and the Chinese Diabetes Society (CDS) CGM Clinical Application Guidelines. Time in range (TIR) indicating the percentage of time that blood glucose was in the range of 3.9-10.0mmol/L. Time in tight range(TITR) represents the percentage of time that the 24-hour blood glucose level is within the range of 3.9-7.8 mmol/L. Based on the original blood glucose data recorded by this system, a number of metrics concerning glycemic variability (GV), including standard deviation (SD), mean amplitude of glycemic excursions (MAGE), mean of daily differences in glucose (MODD), and coefficient of variation (CV) were calculated using the EasyGV Version 9.0R2 provided by Oxford University.

### 2.4 Statistical analysis

The SPSS 26.0 software package was used for statistical analysis. All continuous data underwent normality testing via the Shapiro-Wilk test: Data that passed the Shapiro-Wilk test (P > 0.05) were considered normally distributed, presented as mean ± standard deviation (x ± s), and analyzed using one-way ANOVA for group comparisons. Data that failed the Shapiro-Wilk test (P ≤ 0.05) were identified as skewed, described using medians (interquartile ranges, 25th-75th percentiles), and compared with non-parametric tests (Kruskal-Wallis H test for multiple independent groups, Mann-Whitney U test for two independent groups). The relationship between thyroid function indices (FT3, FT4, TSH) and blood glucose fluctuation parameters (SD, CV, MODD, MAGE) was assessed using Spearman correlation analysis. Multivariate logistic regression analysis was applied to evaluate the association of thyroid hormones with blood glucose fluctuations and DKD. Model 1 was adjusted for age and sex. In accordance with the KDIGO 2020 Clinical Practice Guidelines (19), the following covariates were incorporated into the model 2: duration of diabetes, glycated hemoglobin (HbA1c), hypertension, systolic blood pressure (SBP)/diastolic blood pressure (DBP), lipid profiles, and use of renin-angiotensin-aldosterone system (RAAS) inhibitors/sodium-glucose cotransporter 2 (SGLT2) inhibitors. These variables are explicitly identified as independent risk factors for DKD in the aforementioned guidelines and thus represent “essential basic covariates that must be controlled for” in multivariate regression analyses. To assess the ability of thyroid function indices (FT3, FT4, TSH) to distinguish DKD from non-DKD, we generated ROC curves and calculated AUC with 95% CI; to verify if the FT3-DKD association is consistent across glycemic control levels, we stratified the cohort by HbA1c (HbA1c < 7.0%; HbA1c≥7.0%) and performed multivariate logistic regression in each subgroup. To address multiple comparisons, the Benjamini-Hochberg (BH) method (false discovery rate = 0.05) was applied to Spearman correlation analyses (between thyroid function indices and glycemic variability parameters) and Kruskal-Wallis H tests (comparing glycemic variability parameters across thyroid hormone tertiles), the Bonferroni correction (family-wise error rate = 0.05) was used for multivariate logistic regression analyses (evaluating associations between thyroid hormones and DKD). Statistical significance was set at a two-sided P < 0.05 for all analyses in this study.

## 3 Results

### 3.1 Comparison of clinical characteristics among diabetic kidney disease group and non-diabetic kidney disease group

As shown in **Table 1**, among the 559 euthyroid T2DM patients included in the final cohort, 136 were diagnosed with DKD, resulting in an overall DKD prevalence of 24.3%. Compared with patients without kidney disease, those with kidney disease had no significant differences in glycemic control (HbA1c, TIR, TITR) or blood glucose fluctuation indicators (SD, CV, MODD, MAGE). However, thyroid hormone levels varied between the two groups. The DKD group had significantly lower FT3 levels than the non-DKD group (P<0.01), while no significant differences were found in TSH and FT4 levels between them.

**Table 1:** Clinical characteristics of participants.

Characteristics	Total (n=559)	Non-DKD (n=423)	DKD (n=136)	*P* value
Male n (%)	403 (72.1)	294 (69.5)	109 (75.7)	0.016
Age (years)	55 (46,64)	55 (45, 63)	58 (50, 65.75)	0.012
Diabetes duration (years)	7 (2,13)	6 (2,12)	10 (5,16)	0.000
Smoking n (%)	174 (31.13)	123 (29.08)	51 (37.5)	0.065
Drinking n (%)	143 (25.58)	103 (24.35)	40 (29.41)	0.239
Hypertension n (%)	278 (49.73)	191 (45.15)	87 (63.97)	0.000
SBP (mmHg)	130 (122, 142)	130 (120,140)	135 (125,150.75)	0.000
DBP (mmHg)	80 (74,89)	80 (74,87)	85 (75,92)	0.001
Scr (umol/L)	59 (49,71)	57 (48,66.7)	68.8 (57,89)	0.000
eGFR (ml/min/1.73m²)	106.16 (95.07,116.03)	107.91 (99.01,117.83)	98.82 (75.40,109.74)	0.000
HbA1c (%)	8.7 (7.3,10.1)	8.8 (7.3,10.3)	8.3 (7.2,9.7)	0.087
TIR (%)	71.36 (48.97, 86.27)	71.94 (49.22,86.27)	69.21 (46.12,86.69)	0.57
TITR (%)	33.75 (13.67,57.81)	34.56 (14.27,58.54)	28.35 (13.31,57.45)	0.32
SD (mmol/L)	2.21 (1.65,2.88)	2.19 (1.63,2.90)	2.31 (1.73,2.84)	0.62
CV (%)	0.24 (0.19,0.30)	0.24 (0.19,0.30)	0.24 (0.19,0.30)	0.88
MODD (mmol/L)	1.98 (1.39,2.70)	1.96 (1.35,2.62)	2.06 (1.43,2.78)	0.24
MAGE (mmol/L)	4.15 (3.29,5.30)	4.16 (3.33,5.32)	4.06 (2.96,5.06)	0.19
TSH (mIU/L)	1.77 (1.16,2.6)	1.72 (1.15,2.42)	1.92 (1.19,2.87)	0.07
FT3 (pmol/L)	4.48 (4.19,4.85)	4.52 (4.25,4.86)	4.38 (4.07,4.77)	0.001
FT4 (pmol/L)	11.3 (10.3,12.49)	11.21 (10.30,12.40)	11.6 (10.18,12.65)	0.38
Anti-hypertensive treatment n (%)				
RAAS inhibitors	154 (27.55)	93 (21.99)	61 (44.85)	0.000
Calcium channel blockers	122 (21.82)	75 (17.73)	47 (34.56)	0.000
b-blockers	41 (7.33)	24 (5.67)	17 (12.5)	0.008
Diuretics	23 (4.11)	15 (3.55)	8 (5.89)	0.233
Aspirin therapy	100 (17.89)	70 (16.55)	30 (22.06)	0.158
Statin therapy	186 (33.27)	118 (27.9)	68 (50)	0.000
Anti-diabetic treatment n (%)				
Insulin	321 (57.42)	227 (53.66)	94 (69.12)	0.002
Glucagon like peptide-1	122 (21.82)	95 (22.46)	27 (19.85)	0.522
Metformin	411 (73.52)	317 (74.94)	94 (69.12)	0.181
Sulfonylureas	39 (6.98)	37 (8.75)	2 (1.47)	0.004
Glinides	55 (9.84)	37 (8.75)	18 (13.24)	0.126
α-glucosidase inhibitors	190 (33.99)	153 (36.17)	37 (27.21)	0.055
Thiazolidinediones	29 (5.19)	21 (4.96)	8 (5.88)	0.675
DPP-4 inhibitors	125 (22.36)	8.9 (21.04)	36 (26.47)	0.186
SGLT-2 inhibitors	24 (4.29)	12 (2.84)	12 (8.82)	0.003

Data are expressed as median (interquartile range: 25th to 75th percentile).

HbA1c, glycated hemoglobin A1c; eGFR, estimate glomerular filtration rate; TITR, time in tight range; TIR, time in range; SD, standard deviation of glucose levels CV, coefficient of variation of glucose; MODD, mean of daily differences; MAGE, mean amplitude of glycemic excursions; SBP, systolic blood pressure; DBP, diastolic blood pressure; FT3, free triiodothyronine; FT4, free thyroxine; TSH, thyroid stimulating hormone; eGFR, estimated glomerular filtration rate; DPP-4 inhibitors, Dipeptidyl Peptidase-4 inhibitors; SGLT-2 inhibitors, sodium glucose cotransporter 2 inhibitors; RAAS inhibitors, renin-angiotensin-aldosterone system inhibitors.

### 3.2 Association between thyroid function and glycemic variability

Participants were stratified into tertiles based on FT3 percentiles: T1(low-normal FT3) ≤4.3 pmol/L, n=192; T2(mid-normal FT3): 4.31-4.7 pmol/L, n=181; T3(high-normal FT3): ≥ 4.71 pmol/L, n=186. As demonstrated in **Table 2**, significant differences between group differences emerged in SD, CV, and MODD (P <0.05). However, no statistically significant disparities were observed in glycemic control metrics (HbA1c, TITR and TIR) across the tertiles. Spearman correlation analysis revealed significant inverse associations between FT3 levels and glycemic variability indices (**Table 3**). Specifically, FT3 demonstrated negative correlations with CV (*r_s_* = -0.136, P <0.01), MODD (*r_s_* = -0.093, P <0.05), and SD (*r_s_* = -0.111, P <0.01). No significant associations were found between the two indicators FT4 and TSH and the indicators of glycemic variability.

**Table 2:** Comparison of glycemic variability and prevalence of DKD among the FT3 tertile groups.

Characteristics	T1 (n=192)	T2 (n=181)	T3 (n=186)	*P* value
Male n (%0	138 (72)	125 (69)	140 (75)	0.414
Age (years)	58 (52,68)	55 (46,63)	52 (42.75,60)	0.000
Diabetes duration (years)	9 (4,15)	7 (2,13)	5 (2,10)	0.001
HbA1c (%)	8.7 (7.40,10.4)	9 (7.3,10.30)	8.6 (7.20,10.0)	0.295
TIR (%)	68.05 (45.99, 83.93)	74.19 (55.59,87.75)	71.65 (46.90,86.77)	0.14
TITR (%)	33.49 (14.06, 55.38)	36.98 (14.28, 63.53)	32.28 (12.56, 56.70)	0.462
SD (mmol/L)	2.29 (1.84,3.05)	2.26 (1.57,2.92)	2.06 (1.55,2.71)	0.021
CV (%)	0.25 (0.21,0.31)	0.24 (0.19,0.31)	0.22 (0.18,0.28)	0.002
MODD (mmol/L)	2.13 (1.52,2.95)	1.94 (1.32,2.54)	1.92 (1.36,2.53)	0.039
MAGE (mmol/L)	4.23 (3.34,5.31)	4.31 (3.04,5.50)	3.93 (3.10,5.04)	0.081
DKD (%)	32.3	20.4	19.9	0.006

HbA1c, glycated hemoglobin A1c; TITR, time in tight range; TIR, time in range; SD, standard deviation of glucose levels CV, coefficient of variation of glucose; MODD, mean of daily differences; MAGE, mean amplitude of glycemic excursions; DKD, diabetic kidney disease.

**Table 3:** Correlation of thyroid function parameters with glycemic fluctuation indexes.

Characteristics	SD (mmol/L)		CV (%)		MODD (mmol/L)		MAGE (mmol/L)	
	*r*	*P* value	*r*	*P* value	*r*	*P* value	*r*	*P* value
FT3 (pmol/L)	-0.111	0.009	-0.136	0.001	-0.093	0.03	-0.078	0.071
FT4 (pmol/L)	-0.021	0.621	-0.065	0.126	-0.006	0.89	-0.023	0.599
TSH (mIU/L)	-0.049	0.246	0.779	0.012	0.055	0.082	0.036	0.09

SD, standard deviation of glucose levels CV, coefficient of variation of glucose; MODD, mean of daily differences; MAGE, mean amplitude of glycemic excursions; FT3, free triiodothyronine; FT4, free thyroxine; TSH, thyroid stimulating hormone.

Patients were divided into three groups based on FT4 tertiles: T1 (FT4 ≤10.7, n=191), T2 (10.71<FT4<12.1, n=185), and T3 (FT4≥12.11, n=183). As shown in **Table 4**, no significant differences were observed in blood glucose fluctuation indicators (SD, CV, MODD, MAGE) among the three groups. However, significant differences in HbA1c levels were found between the groups.

**Table 4:** Comparison of glycemic variability and prevalence of DKD among the FT4 tertile groups.

Characteristics	T1 (n=191)	T2 (n=185)	T3 (n=183)	*P* value
Male n (%)	141 (73.8)	133 (71.9)	129 (70.5)	0.771
Age (years)	55.67 (49,63)	53.25 (42,64)	55.49 (47,66)	0.12
Diabetes duration (years)	8.63 (3,13)	8.26 (3,12)	8.65 (2,14.25)	0.997
HbA1c (%)	8.49 (7.1,9.63)	9.0 (7.5,10.4)	9.17 (7.48,10.55)	0.015
TIR (%)	74.09 (52.46,86.94)	66.64 (46.89,85.41)	73.08 (44.77,86.0)	0.328
TITR (%)	38.32 (15.21,58.4)	31.90 (12.67,57.58)	31.98 (12.71,60.10)	0.462
SD (mmol/L)	2.19 (1.64,2.84)	2.25 (1.76,2.94)	2.19 (1.57,2.84)	0.361
CV (%)	0.25 (0.19,0.31)	0.26 (0.20,0.30)	0.24 (0.18,0.30)	0.239
MODD (mmol/L)	2.17 (1.38,2.68)	2.26 (1.46,2.72)	2.22 (1.33,2.72)	0.708
MAGE (mmol/L)	4.27 (3.31,5.16)	4.38 (3.16,5.42)	4.49 (3.28,5.31)	0.98
DKD (%)	46 (24.1)	42 (22.7)	48 (26.2)	0.73

HbA1c, glycated hemoglobin A1c; TITR, time in tight range; TIR, time in range; SD, standard deviation of glucose levels CV, coefficient of variation of glucose; MODD, mean of daily differences; MAGE, mean amplitude of glycemic excursions; DKD, diabetic kidney disease.

Patients were categorized into three groups based on TSH tertiles: T1 (TSH ≤ 1.34, n=187), T2 (1.35<TSH<2.19, n=186), and T3 (TSH≥2.20, n=186). As shown in **Table 5**, no significant differences were found in blood glucose fluctuation indicators (SD, CV, MODD, MAGE) among the three groups. However, significant differences in HbA1c levels were observed between the groups (P<0.05), with HbA1c decreasing as TSH increased.

**Table 5:** Comparison of glycemic variability and prevalence of DKD among the TSH tertile groups.

Characteristics	T1 (n=187)	T2 (n=186)	T3 (n=186)	*P* value
Male n (%)	148 (79.1)	132 (71)	123 (66.1)	0.018
Age (years)	54.8 (45, 64)	54.75 (46.75, 63)	54.87 (47, 64.25)	0.958
Diabetes duration (years)	8.23 (3,12)	8.73 (2,14)	8.59 (2,14)	0.974
HbA1c (%)	9.17 (7.73,10.4)	8.80 (7.25,10.2)	8.68 (7.1,9.9)	0.038
TIR (%)	71.02 (47.3,85.53)	70.01 (49, 86.76)	74.15 (47.74, 88)	0.388
TITR (%)	32.71 (10.83, 57.75)	32.24 (14.88, 55.26)	37.44 (14.50, 61.40)	0.497
SD (mmol/L)	2.26 (1.78,2.90)	2.20 (1.62,2.85)	2.15 (1.59,2.88)	0.415
CV (%)	0.25 (0.20,0.30)	0.26 (0.19,0.30)	0.25 (0.19,0.30)	0.762
MODD (mmol/L)	2.28 (1.46,2.84)	2.26 (1.33,2.7)	2.1 (1.39,2.5)	0.192
MAGE (mmol/L)	4.53 (3.49, 5.53)	4.34 (3.1,5.05)	4.28 (3.15,5.23)	0.058
DKD (%)	40 (21.4)	42 (22.6)	54 (29)	0.181

HbA1c, glycated hemoglobin A1c; TITR, time in tight range; TIR, time in range; SD, standard deviation of glucose levels CV, coefficient of variation of glucose; MODD, mean of daily differences; MAGE, mean amplitude of glycemic excursions; DKD, diabetic kidney disease.

### 3.3 The relationship between thyroid hormones and the prevalence of diabetic kidney disease

**Table 6** shows the association between thyroid hormone levels and DKD based on multivariate logistic regression when the thyroid hormone is considered as a continuous variable. **Table 7** shows the association in different thyroid hormone tertiles. Model 1 was adjusted for age and sex, Model 2 was adjusted for age, sex, diabetic duration, fasting blood-glucose, HbA1c, systolic blood pressure, diastolic blood pressure, total cholesterol, triglycerides, HDL-C, LDL-C, β-blockers, statins, RAAS inhibitors, and SGLT2 inhibitor usage. It was found that TSH were identified as risk factors for DKD, while FT3 emerged as a protective factor.

**Table 6:** Association of thyroid status with diabetic kidney disease.

Thyroid status	Diabetic kidney disease			
	Model 1		Model 2	
	OR (95%CI)	*P*-Value	OR (95%CI)	*P*-Value
FT3 (pmol/L)	0.536 (0.331-0.867)	0.011	0.428 (0.248-0.738)	0.002
FT4 (pmol/L)	1.123 (0.975-1.294)	0.106	1.146 (0.976-1.347)	0.097
TSH (mIU/L)	1.275 (1.065-1.527)	0.008	1.322 (1.084-1.611)	0.006

Evaluating the risk of DKD when the thyroid hormone is considered as a continuous variable. Model 1: adjusted for age and sex. Model2: Model 1 plus adjusted for systolic blood pressure, diastolic blood pressure, total cholesterol, triglycerides, HDL-C, LDL-C, β-blockers, statins, RAAS inhibitors, and SGLT2 inhibitor usage.

**Table 7:** Association of thyroid status with diabetic kidney disease.

Thyroid status	Diabetic kidney disease			
	Model 1	Model 2		
	OR (95%CI)	*P*-Value	OR (95%CI)	*P*-Value
FT3 (pmol/L)				
1^st^tertile	1.000 (referent)		1.000 (referent)	
2^nd^tertile	0.598 (0.368-0.971)	0.038	0.545 (0.319-0.931)	0.026
3^rd^tertile	0.548 (0.333-0.904)	0.019	0.445 (0.254-0.782)	0.005
FT4 (pmol/L)				
1^st^tertile	1.000 (referent)		1.000 (referent)	
2^nd^tertile	1.068 (0.651-1.752)	0.794	0.975 (0.566-1.678)	0.927
3^rd^tertile	1.280 (0.784-2.088)	0.324	1.319 (0.762-2.284)	0.323
TSH (mIU/L)				
1^st^tertile	1.000 (referent)		1.000 (referent)	
2^nd^tertile	1.221 (0.736-2.023)	0.439	1.076 (0.613-1.887)	0.799
3^rd^tertile	1.785 (1.090-2.924)	0.021	1.801 (1.049-3.092)	0.033

Evaluating the risk of DKD when the thyroid hormone is considered as different tertiles. Model 1: adjusted for age and sex. Model2: Model 1 plus adjusted for systolic blood pressure, diastolic blood pressure, total cholesterol, triglycerides, HDL-C, LDL-C, β-blockers, statins, RAAS inhibitors, and SGLT2 inhibitor usage.

**Figure 1:**
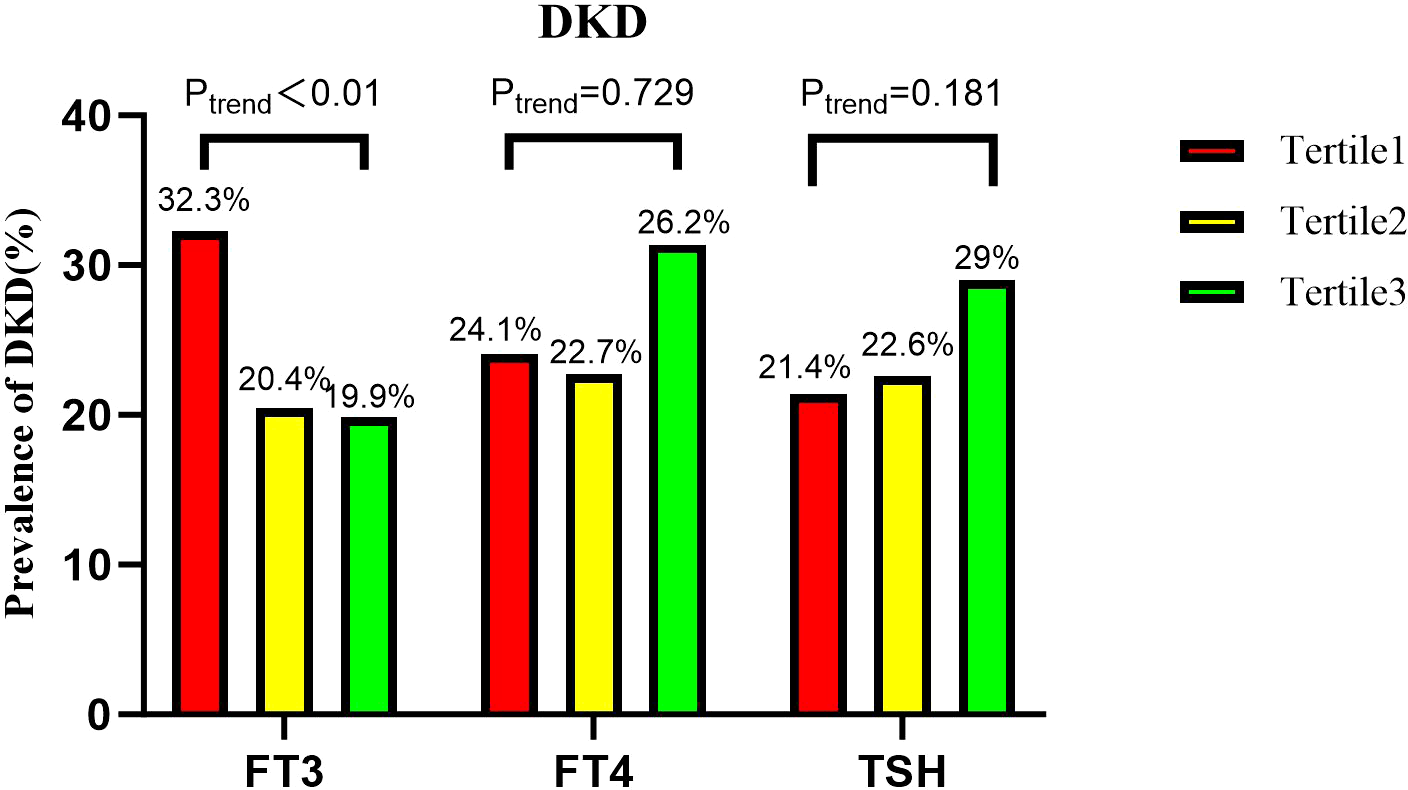
Prevalence of DKD among tertiles based on FT3, FT4, and TSH levels.

**Figure 1** shows the prevalence of DKD in different tertiles of thyroid hormone. There was a statistically significant difference in the prevalence of DKD among FT3 groups (P < 0.01). The high FT3 group had a lower incidence of diabetic kidney disease compared to the low FT3 group. The incidence rates of DKD across the FT3 tertiles (T1, T2, and T3) were 32.3%, 20.4%, and 19.9%, respectively (P < 0.01). There was no significant difference in the prevalence of DKD between the TSH and FT4 tertiles.

### 3.4 Discriminative ability of thyroid function indices for DKD (ROC curve analysis)

**Figure 2:**
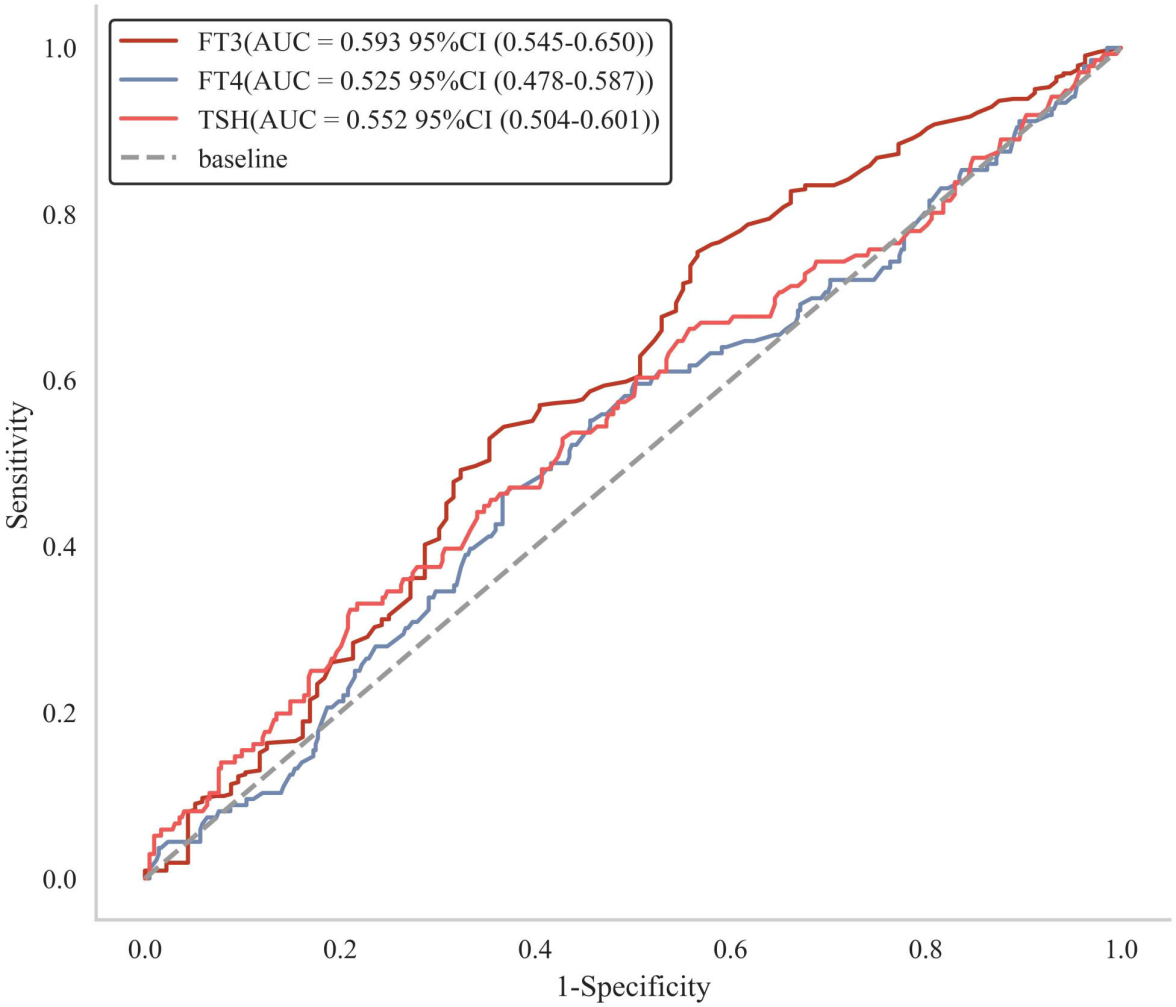
ROC curves of thyroid function indices for discriminating DKD in euthyroid T2DM.

As shown in **Figure 2** and **Table 8**, among the three thyroid hormones: FT3 exhibited the strongest discriminative ability, with an AUC of 0.593 (95% CI: 0.548–0.646). The optimal cutoff value for FT3 was 4.25 pmol/L, corresponding to a sensitivity of 0.754, specificity of 0.434, and accuracy of 0.641. FT4 and TSH showed limited discriminative value, with AUCs of 0.525 (95% CI: 0.469–0.584) and 0.552 (95% CI: 0.498–0.614), respectively. Their optimal cutoffs were 11.84 pmol/L (FT4) and 2.660 mIU/L (TSH), with Youden indices of 0.097 and 0.113 (both <0.2, indicating weak diagnostic performance).

**Table 8:** ROC Curve Metrics of Thyroid Function Indices for Discriminating DKD in Euthyroid T2DM.

Feature	N	AUC (95% CI)	Sensitivity (95% CI)	Specificity (95% CI)	Youden Index (95% CI)	Optimal Cutoff (Unit)	Accuracy (95% CI)
TSH (mIU/L)	559	0.552 (0.498-0.614)	0.331 (0.274-0.735)	0.783 (0.433-0.826)	0.113 (0.077-0.223)	2.660 (mIU/L)	0.625 (0.484-0.742)
FT4 (pmol/L)	559	0.525 (0.469-0.584)	0.463 (0.262-0.669)	0.634 (0.461-0.794)	0.097 (0.045-0.198)	11.840 (pmol/L)	0.564 (0.409-0.709)
FT3 (pmol/L)	559	0.593 (0.548-0.646)	0.754 (0.493-0.840)	0.434 (0.364-0.738)	0.188 (0.144-0.269)	4.250 (pmol/L)	0.646 (0.530-0.738)

FT3, free triiodothyronine; FT4, free thyroxine; TSH, thyroid stimulating hormone; CI, confidence interval.

### 3.5 Stratified analyses

As shown in **Table 9**, in poorly controlled T2DM (HbA1c ≥7.0%), the adjusted OR for FT3 and DKD is 0.406 (95% CI: 0.216–0.765, P = 0.005), confirming a significant negative association (lower FT3 correlates with higher DKD risk) in this subgroup. This aligns with the total cohort result, as expected for a population with more severe disease. In well-controlled T2DM (HbA1c <7.0%), although the adjusted OR (0.861, 95% CI: 0.208–3.558, P = 0.836) is not statistically significant, this is likely due to small sample size (n=109, only 20% of the total cohort) rather than absence of association. The direction of the OR (0.861 < 1) remains consistent with the total cohort and poorly controlled subgroup indicating a trend toward lower DKD risk with higher FT3, even in well-controlled T2DM. Across all subgroups (total, well-controlled, poorly controlled), the OR (crude and adjusted) is consistently <1.

**Table 9:** Stratified Analysis of the Association Between FT3 and DKD by HbA1c.

Stratification	N	OR	95%CI	*P*-value	N (adjusted)	OR (adjusted)	95%CI (adjusted)	*P*-value (adjusted)
Total	559	0.527	(0.336,0.828)	0.005	549	0.428	(0.248,0.738)	0.002
HbA1c (%)								
<7	109	0.517	(0.181,1.480)	0.219	109	0.861	(0.208,3.558)	0.836
≥7	440	0.558	(0.335,0.929)	0.025	430	0.406	(0.216,0.765)	0.005

HbA1c, glycated hemoglobin A1c; CI, confidence interval.

## 4 Discussion

T2DM has shown an upward incidence trend globally, and the hypothalamic-pituitary-thyroid axis dysfunction is frequently observed in this population, leading to abnormal thyroid hormone levels. Thyroid hormones play crucial roles in renal growth, GFR regulation, and sodium balance maintenance. Hypothyroidism can reduce β-adrenergic sensitivity, decrease renin release, and ultimately impair renal self-regulation (20). Notably, even euthyroid T2DM patients may have subclinical thyroid microstructure damage associated with blood glucose levels and insulin resistance, while research on glycemic variability in this subgroup remains limited.

In this study, compared with non-DKD patients, serum FT3 levels were lower in DKD patients. All patients wore a continuous glucose monitor, and blood glucose fluctuation-related indicators were analyzed, including CV, SD, MODD, and MAGE. The blood glucose fluctuation coefficient, namely CV, reflects the unstable state of blood glucose levels between the high and low fluctuation points. The SD of glucose levels is the standard deviation of the daily glucose measurements, reflecting the overall divergence from the mean blood glucose value, i.e., blood glucose fluctuation. MAGE is an indicator for assessing daily blood glucose fluctuations. MODD evaluates the degree of interday blood glucose fluctuations and reflects the reproducibility of daily blood glucose levels. MODD on a specific date reflects the fluctuation degree of glucose values between that day and the previous day. This study found that DKD patients had significantly lower serum FT3 levels than non-DKD patients (4.38 vs. 4.52 pmol/L, *P* = 0.001; **Table 1**). CGM analysis revealed negative correlations between FT3 and glycemic variability indices (CV: *r* = -0.136, *P* = 0.001; SD: *r* = -0.111, *P* = 0.009; MODD: *r* = -0.093, *P* = 0.03; **Table 3**). Multivariate logistic regression further confirmed low FT3 (adjusted OR = 0.428, 95%CI=0.248-0.738, *P* = 0.002) and high thyroid-stimulating hormone (TSH) (adjusted OR = 1.322, 95%CI=1.084-1.611, *P* = 0.006) as independent DKD risk factors (**Table 6**).

Regarding the limitation of study participants, since the participants of this study were inpatients from a tertiary hospital, there may be an overrepresentation of patients with more severe disease or poor glycemic control, which could further compromise the generalizability of the study results. To address this concern, we conducted a stratified analysis by HbA1c levels. The results showed that in the subgroup of T2DM patients with poor glycemic control (HbA1c ≥7.0%), there was a significant negative association between FT3 and DKD (the lower the FT3 level, the higher the DKD risk), which was consistent with the total cohort analysis. In contrast, the adjusted OR in the well-controlled subgroup (HbA1c <7.0%) was not statistically significant, but this was attributed to the small sample size (n=109, 20% of the total cohort) rather than no association. Notably, the consistent direction of OR (<1) across all subgroups confirmed that the FT3-DKD association was genuine, ruling out the influence of selection bias related to severe T2DM overrecruitment.

Importantly, our study revealed differential associations of FT3, FT4, and TSH with DKD, which can be explained by their distinct biological roles in renal physiology and T2DM pathogenesis. FT3, as the biologically active thyroid hormone, exerts direct renal protective effects via three key pathways (1): enhancing pancreatic β-cell function and insulin sensitivity to reduce glycemic fluctuations (21–23) (2), activating SIRT1 to inhibit renal inflammation and fibrosis (24), and (3) stimulating endothelial nitric oxide synthase (eNOS) to preserve renal microvascular function (25, 26). These effects are dependent on FT3’s high affinity for thyroid hormone receptors (TRs) in renal tissues, an attribute that is lacking in FT4. As the major circulating thyroid hormone, FT4 acts primarily as a precursor to FT3; however, hyperglycemia and inflammation in T2DM downregulate 5’-deiodinase (the enzyme converting FT4 to FT3) (27), reducing renal FT3 availability and rendering FT4 biologically irrelevant to DKD. In contrast, TSH lacks renal receptors (20) and does not directly modulate renal function. Its association with increased DKD risk likely reflects indirect effects, such as linking to insulin resistance (28) or serving as a marker of subtle thyroid axis dysfunction (e.g., reduced FT3 bioavailability). Together, these mechanisms explain why only FT3 emerged as a protective factor and TSH as a secondary risk factor, while FT4 showed no significant association with DKD.

Studies have shown that among diabetic patients with similar HbA1c levels, those with greater fluctuations in blood glucose have a higher risk of developing chronic complications (29). The possible mechanism is that increased volatility of blood glucose can amplify oxidative stress, promote the generation and accumulation of advanced glycation end products and lipid oxidation end products in the body, and thereby increase the risk of chronic complications. In a cross-sectional study of middle-aged and elderly Chinese people, the FT3 levels within the normal range were significantly negatively correlated with urinary microalbumin (30). Animal studies have confirmed that supplementation with FT3 can significantly improve proteinuria in mice with type 2 diabetic kidney disease, reduce the accumulation of collagen in the renal cortex, alleviate the dilation of glomerular mesangial matrix, enhance the activity of PI3K in the kidneys, lower blood sugar, and protect renal function (31). In a prospective study of 104,633 Koreans by Zhang et al. (32), high-normal TSH and low-normal FT3 in euthyroid individuals were moderately associated with increased chronic kidney disease risk. Research confirms a positive link between GFR and FT3. Chronic kidney disease patients usually present with hypothyroidism rather than hyperthyroidism. Studies also show that the prevalence of hypothyroidism increases with the decline of renal function, and thyroid hormone replacement therapy can significantly improve the renal function of patients with DKD (28, 33).

There is a close bidirectional interaction between glucose metabolism and thyroid function: On one hand, glucose metabolism disorders directly affect thyroid function—not only may they impair the iodine uptake capacity of thyroid follicles, but also inhibit the activity of 5’-deiodinase in peripheral tissues, thereby reducing the level of triiodothyronine(T3); meanwhile, in diabetic patients, the levels of cytokines such as interleukin-1 (IL-1) and interleukin-6 (IL-6) are elevated, and these inflammatory factors further interfere with the synthesis, transport, and release of TSH and T3 (27). On the other hand, abnormal thyroid function also exerts a countereffect on glucose metabolism. This effect is particularly evident when the levels of T3 and FT3 decrease, it affects the normal metabolism of glucose, lipids, water, and electrolytes, ultimately exacerbating glucose metabolism disorders and forming a vicious cycle of “abnormal glucose metabolism - impaired thyroid function”.

It is particularly important to emphasize that due to the cross-sectional design of this study, it can only observe the associations between variables at a single time point and cannot clarify the causal relationship and direction of action between variables. This limitation hinders our ability to determine, in euthyroid T2DM patients, whether thyroid hormone changes precede DKD or glycemic instability.

or are secondary to early renal impairment or DKD-related metabolic disorders. Future prospective studies are therefore needed to clarify this causal ambiguity. Specifically, both directions of action are possible: On one hand, the progression of DKD itself may have a subtle impact on thyroid hormone metabolism, as it is known that CKD interferes with iodine handling, thyroid hormone synthesis, and the conversion of thyroxine (T4) to T3 in peripheral tissues (20, 33)., which may lead to decreased FT3 levels or increased TSH levels even within the euthyroid range; On the other hand, as discussed in the previous mechanism analysis, low FT3 levels may also be directly involved in the pathogenesis of DKD through pathways such as impairing insulin secretion, inducing endothelial dysfunction, or enhancing inflammatory responses. Similarly, the negative correlation between FT3 and glycemic variability may also reflect a bidirectional effect: low FT3 may exacerbate glycemic variability by disrupting insulin sensitivity or pancreatic β-cell function; in turn, long-term glycemic instability may impair thyroid hormone metabolism through oxidative stress or cytokine-mediated pathways, further amplifying the abnormal association between the two (27).

It is also important to note the potential influence of medication use on the observed associations. Although we excluded patients with a history of thyroid medication use (e.g., levothyroxine) to avoid direct interference with thyroid hormone levels, the use of anti-diabetic and anti-hypertensive drugs may still indirectly affect glucose fluctuations and thyroid function. To mitigate these confounding effects, we included key medication use as adjustment variables in the multivariate logistic regression model (Model 2). However, limitations remain: First, we did not collect detailed information on medication dosage, treatment duration, and adherence, which may affect the accuracy of confounding adjustment. Second, the use of combination anti-diabetic drugs was common in the cohort, and the interactive effects of multiple drugs on glucose fluctuations and thyroid function could not be fully disentangled.

Based on the limitations of this study and existing evidence, future research can further advance exploration from multiple dimensions: First, prospective cohort studies should be conducted to clarify whether a baseline low-normal FT3 level can predict the onset of DKD. Second, greater attention should be paid to controlling medication-related confounding factors. On one hand, detailed medication information (including dosage, duration, adherence, and combination regimens) should be collected, and more sophisticated statistical methods such as propensity score matching should be adopted to reduce the interference of medication use on research results. On the other hand, interventional trials with standardized medication regimens or drug washout periods can be designed. These trials not only verify whether FT3 supplementation can mitigate the progression of DKD but also help clarify the independent associations among thyroid hormones, glycemic variability, and DKD. Finally, multicenter study designs should be adopted, incorporating community-dwelling patients and diverse ethnic groups to validate the applicability of the FT3 cutoff value across different populations, thereby further enhancing the generalizability of research conclusions and their clinical translational value.

## 5 Conclusion

In conclusion, this cross-sectional study revealed that in euthyroid type 2 diabetics, those with low-normal FT3 levels exhibit greater blood glucose fluctuations and a higher incidence of DKD. This suggests that low-normal FT3 within the normal range could serve as a potential risk factor for blood glucose instability and DKD development, potentially informing DKD prevention strategies. However, the causal relationship and mechanism between FT3 and DKD require further prospective studies for confirmation.

## Data Availability

The raw data supporting the conclusions of this article will be made available by the authors, without undue reservation.
